# Lipid-Lowering Therapy after Acute Coronary Syndrome

**DOI:** 10.3390/jcm13072043

**Published:** 2024-04-01

**Authors:** Edita Pogran, Achim Leo Burger, David Zweiker, Christoph Clemens Kaufmann, Marie Muthspiel, Gersina Rega-Kaun, Alfa Wenkstetten-Holub, Johann Wojta, Heinz Drexel, Kurt Huber

**Affiliations:** 1 3rd Medical Department for Cardiology and Intensive Care Medicine, Klinik Ottakring, 1160 Vienna, Austria; 2 Medical Faculty, Sigmund Freud University, 1020 Vienna, Austria; 3 5th Medical Department with Endocrinology, Rheumatology and Acute Geriatrics, Klinik Ottakring, 1160 Vienna, Austria; gersina.rega-kaun@gesundheitsverbund.at (G.R.-K.);; 4 Ludwig Boltzmann Institute for Cardiovascular Research, 1090 Vienna, Austria; johann.wojta@meduniwien.ac.at; 5 Core Facilities, Medical University of Vienna, 1090 Vienna, Austria; 6 Vorarlberg Institute for Vascular Investigation and Treatment (VIVIT), Carinagasse 47, 6800 Feldkirch, Austria

**Keywords:** lipid-lowering therapy, acute coronary syndrome, cardiovascular disease, secondary prevention, statins, ezetimibe, PCSK9-inhibitor, bempedoic acid, inclisiran

## Abstract

Achieving guideline-recommended low-density lipoprotein cholesterol (LDL-C) targets remains a significant challenge in clinical practice. This review assesses the barriers to reaching LDL-C goals and explores the potential solutions to these issues. When aiming for the recommended LDL-C goal, strategies like “lower is better” and “strike early and strong” should be used. The evidence supports the safety and efficacy of intensive lipid-lowering therapy post-acute coronary syndrome (ACS), leading to improved long-term cardiovascular health and atherosclerotic plaque stabilization. Despite the availability of effective lipid-lowering therapies, such as high-intensity statins, ezetimibe, the combination of both, bempedoic acid, and proprotein convertase subtilisin/kexin type 9 (PCSK9) inhibitors, a substantial proportion of patients do not meet their LDL-C targets. Contributing factors include systemic healthcare barriers, healthcare provider inertia, patient non-adherence, and statin intolerance. Statin intolerance, often rather statin reluctance, is a notable obstacle due to perceived or expected side effects, which can lead to discontinuation of therapy. In conclusion, while there are obstacles to achieving optimal LDL-C levels post-ACS, these can be overcome with a combination of patient-centric approaches, clinical vigilance, and the judicious use of available therapies. The safety and necessity of reaching lower LDL-C goals to improve outcomes in patients post-ACS are well-supported by current evidence.

## 1. Introduction

Cardiovascular disease (CVD) is the leading cause of death worldwide and a major contributor to disability [1,2]. The epidemiology of CVD and acute coronary syndrome (ACS) underlines a grim reality: CVD claims a life every 38 s in the United States alone, and more than 7 million individuals are diagnosed with ACS annually worldwide [3]. The financial implications are staggering, with direct costs in the U.S. surpassing USD 216 billion, and projections suggesting a surge to USD 749 billion by 2035. These costs reflect not only healthcare expenditures but also the indirect burden of decreased productivity and quality of life [2].

Risk factors for CVD are well-known and largely modifiable, including diabetes, hypertension, dyslipidemia, and lifestyle choices such as smoking and physical inactivity [1,4,5]. It is known that over 90% of CVD risk is attributable to such factors, suggesting a significant potential for prevention [6]. Research into hyperlipidemia, a major risk factor, has yielded numerous treatment options and advances, with a strong emphasis on the importance of lowering low-density lipoprotein cholesterol (LDL-C) to prevent recurrent cardiovascular events.

Aggressive control of modifiable risk factors could prevent up to 80% of premature CVD-related deaths, underlining the crucial role of healthcare providers in maximizing prevention efforts. Improvements in community health could prevent millions of major CVD events annually [7]. With cholesterol levels remaining high among adults, and LDL-C identified as a causal factor in atherosclerotic cardiovascular disease (ASCVD), reducing LDL-C levels is recognized as a primary therapeutic goal, particularly for those at the highest risk of future events [8].

The routine assessment of lipid profiles in clinical practice, which includes total cholesterol, LDL-C, HDL-c (high-density lipoprotein cholesterol), and triglycerides, offers a general snapshot of lipid metabolism status. However, this traditional lipid panel may not fully capture the complexity of lipid abnormalities, particularly in the context of oxidative stress and its impact on atherosclerosis. For example, LDL particles can be altered by oxidative stress, increasing their atherogenic potential. Yet, these modified lipoproteins, such as oxidized LDL, are not typically measured in standard practice, although they may contribute significantly to CVD progression in patients with diabetes mellitus. Therefore, while LDL cholesterol concentration remains a critical target for lipid-lowering therapy, its role as a marker should be considered within the broader context of individual CVD risk assessment. This includes recognizing the potential contributions of modified lipoproteins and oxidative stress markers, which may offer additional insights into cardiovascular risk, particularly in patients with diabetes mellitus where such alterations are more pronounced. This remains a topic for further research.

Furthermore, in the aftermath of an ACS event, the risk of subsequent episodes remains significant, with a 30% chance of another occurrence within two years [9]. This review will thus focus on the strides made in hyperlipidemia research and the diverse treatment landscape available to combat this preventable yet persistent contributor to CVD and ACS.

## 2. Current Recommendations on Lipid-Lowering Therapy

The 2019 guidelines from the European Society of Cardiology (ESC) on the treatment of patients with dyslipidemia recommended an LDL-C reduction of ≥50% from baseline values and a treatment target of <55 mg/dL (1.4 mmol/L) in patients at very high risk, including patients with documented ASCVD and severe chronic kidney disease (eGFR < 30 mL/min/1.73 m^2^) or patients with diabetes mellitus and target organ damage, respectively. Importantly, an even greater LDL-C reduction below 40 mg/dL (1.0 mmol/L) had to be considered in patients with ASCVD and a second vascular event within two years while taking maximally tolerated statin-based therapy. Initiation of lipid-lowering therapy was recommended in a stepwise approach, starting with high-intensity statin therapy and treatment intensification with ezetimibe after 4–6 weeks if the LDL-C target is missed (class I recommendation). A further escalation with a proprotein convertase subtilisin/kexin type-9 (PCSK9) inhibitor was recommended after an additional period of 4–6 weeks if the treatment target still failed to be achieved [10]. Finally, icosapent ethyl, at a dose of 2 g b.i.d., may be prescribed in combination with a statin in patients with ACS and triglyceride levels of 135–499 mg/dL (1.5–5.6 mmol/L) despite statin treatment [10,11].

The latest recommendations from the ESC on the treatment of dyslipidemia were embedded in the 2023 guidelines on the treatment of ACS. These guidelines still recommend (class I) a stepwise approach starting with high-potency high-intensity statin [12]. However, in consideration of the emerging treatment strategy of “strike early and strong”, a more rapid treatment initiation of lipid-lowering therapy has been applied [2,3]. In previously lipid-lowering treatment-naive patients with ACS, immediate combination therapy with high-intensity statin and ezetimibe may be considered (class IIb recommendation) in order to accelerate LDL-C treatment target achievement. This recommendation reflects a clinical consensus statement of the Association for Acute CardioVascular Care from 2022, suggesting early and strong LDL-C reduction post-ACS by starting a combination of high-intensity therapy and ezetimibe [13]. Escalation with a PCSK9 inhibitor is recommended if treatment targets fail to be achieved [12,13].

While this treatment approach with a more rapid initiation of lipid-lowering therapy has gained importance and recognition, former and current guidelines from the American Heart Association and the American College of Cardiology still recommend a stepwise initiation of lipid therapy [5,14].

## 3. Real-World Data

The Da Vinci and Santorini studies offer crucial insights into the practical application of lipid-lowering therapy (LLT) in patients post-ACS, with a focus on real-world data [8,15].

The Da Vinci study, an extensive 18-country cross-sectional observational study carried out between June 2017 and November 2018, enrolled 5888 patients across Europe. These patients were already on LLT for primary or secondary prevention. The study’s primary aim was to evaluate the achievement of LDL-C goals based on the 2016 and 2019 ESC/European Atherosclerosis Society (EAS) guidelines. The results were sobering: only 54% of the patients managed to achieve their LDL-C goals as per the 2016 guidelines, and even fewer, about 33%, reached the more stringent 2019 guideline-recommended targets. The study also reported that high-intensity statin monotherapy was used by 20% of the very high-risk primary prevention patients and 38% of secondary prevention patients, with proportionally higher use among those being managed for coronary disease (51%) than for peripheral (39%) or cerebral (40%) diseases [15].

The Santorini study, a prospective observational non-interventional study conducted from 17 March 2020 to 11 February 2021, focused on lipid management post the 2019 update of the ESC/EAS guidelines. The study included patients at high and very high cardiovascular risk across Europe. The main finding of the study was that only 20.7% of patients with ASCVD reached the recommended LDL-C goal by 2019 ESC/EAS guidelines. Moreover, 21.0% of very high-risk patients and 23.5% of high-risk patients did not have any LLT at all [8].

Furthermore, another significant finding from the study was that physicians frequently underestimated the very high risk in patients, leading to false risk stratification. According to physicians, 29.2% were classified as high cardiovascular risk and 70.8% as very high risk. However, when the cardiovascular risk was assessed centrally, only 6.5% of patients were at high risk and 91.0% of patients were in the very high-risk group [8].

Moreover, Zuin et al. investigated seven studies reporting real-world data from more than 36,000 patients after ACS in Europe. Overall, only 12.1% of the patients achieved the recommended LDL-C levels [16]. These results are in agreement with the most recent multinational European survey study of ACS patients (between 2021 and 2022), which showed that only 25.7% of ACS patients on combination therapy (statin + ezetimibe) and just 16.5% of ACS patients on high-intensity statin monotherapy reached the ESC LDL-C reduction target [17].

In the United States, data from 10,589 patients recently discharged with an ACS event between 2013 and 2019 showed that 49% filled a prescription for a high-intensity statin at discharge, but only 36% were adherent at 1 year. Adherence was strongly associated with clinical characteristics such as ACS type and baseline LDL-C values, with disparities observed in fill rates and adherence based on age, sex, and race/ethnicity [18].

In a study performed in India, it was reported that out of 575 patients included in the study, only 20.9% managed to achieve a target LDL-C of <55 mg/dL (1.4 mmol/L) after one year, despite being on high-intensity statin therapy [19].

In summary, despite the clear guidelines and evidence supporting the benefits of LLT, there remains a considerable gap in the actual management of dyslipidemia in real-world settings (Table 1). These findings underscore the need for a more aggressive approach to lipid management and improved implementation strategies at both the patient and healthcare provider levels.

## 4. The Lower the Better

Statins are the cornerstone in the management of dyslipidemia, not only but particularly for patients at very high cardiovascular risk. Numerous studies have demonstrated that a lower level of LDL-C correlates with a reduced risk of cardiovascular events, giving rise to the paradigm “the lower the better”.

There is a sufficient number of studies available suggesting the benefit of statin treatment in primary prevention [22,23,24,25,26]. Moreover, secondary prevention trials also support the benefits of statins. The Scandinavian Simvastatin Survival Study (4S) was the first large clinical trial that demonstrated not only reductions in cardiovascular events but also an increase in survival in secondary prevention, with a 30% relative risk reduction in all-cause mortality in the simvastatin group [27]. The MIRACL and TNT studies further emphasized the importance of intensive LDL-C lowering. In the MIRACL study, atorvastatin initiated shortly after an acute MI led to a significant relative risk reduction of 16% of the primary endpoint [28]. The TNT study demonstrated that atorvastatin 80 mg in comparison with atorvastatin 10 mg significantly reduced the risk of major cardiovascular outcomes by 22% in patients with stable CHD [29]. Moreover, meta-analyses of 26 randomized trials comparing more versus less intensive statin regimens showed that 1.0 mmol/L LDL-C reduction decreased all-cause mortality by 10%, largely reflecting a significant reduction in deaths due to coronary heart disease and other cardiac causes [30].

The IMPROVE-IT trial represents a landmark in combination therapy, showing that adding ezetimibe to statins further lowered LDL-C levels (from a median of 70 mg/dL (1.8 mmol/L) to 54 mg/dL (1.4 mmol/L) and still improved cardiovascular outcomes, with a relative risk reduction of 6.7% after 7 years. This study included 18,144 patients with ACS and highlighted the additive benefits of combining ezetimibe with statins [31].

PCSK9 inhibitors have significantly impacted the field of lipid management, further cementing the “lower the better” approach in lowering LDL-C levels and associated cardiovascular risk. The FOURIER trial is a cornerstone study that demonstrated the effectiveness of PCSK9 inhibition in patients with stable coronary artery disease. The PCSK9 inhibitor evolocumab lowered LDL-C by 59% to a median of 30 mg/dL (0.8 mmol/L), which led to a 15% risk reduction of the primary endpoint—a composite of cardiovascular death, MI, stroke, hospitalization for unstable angina, or coronary revascularisation—over a median follow-up of 2.2 years [32].

Not only very low LDL-C levels but also long-term LDL-C lowering bring enormous benefits. Evolocumab was associated with persistently low rates of adverse events over 8 years and led to further reductions in cardiovascular events compared with delayed treatment initiation, as suggested by FOURIER-OLE study [33].

The ODYSSEY study results align with FOURIER findings in patients after an ACS, where alirocumab treatment decreased LDL-C by 63% and was associated with a 15% reduction in the risk of the primary endpoint—composed of death from coronary heart disease, non-fatal MI, fatal or non-fatal ischemic stroke, or unstable angina requiring hospitalization—after a median follow-up of 2.8 years [34]. Furthermore, both studies reported that the total number of cardiovascular events—including recurrent events—prevented by aggressive LDL-C lowering was approximately double that of the first occurrence of an ASCVD primary endpoint, indicating that the benefits of profound LDL-C lowering extend beyond the prevention of first ASCVD events [35,36].

Supporting the concept of even lower LDL-C targets, the FOURIER trial found that patients achieving LDL-C levels below 20 mg/dL (0.5 mmol/L) after four weeks of evolocumab treatment had the lowest risk for ischemic events in the entire study population without an increase in adverse events [37]. Similarly, a propensity score-matched analysis of the ODYSSEY Outcomes study reported that patients achieving an LDL-C of <25 mg/dL (0.65 mmol/L) had a particularly low risk of major adverse cardiovascular events (MACEs) similar to that of patients who achieved LDL-C levels of 25–50 mg/dL (0.65–1.3 mmol/L) without excess risk of hemorrhagic stroke or dementia [38]. These results were further supported by a recent meta-analysis, including 10 randomized trials and over 38,000 subjects, which showed that very low LDL-C levels on intensive lipid-lowering treatments are not associated with any adverse event and maintain a persistent reduction in cardiovascular events [39].

The GLAGOV trial further substantiated the benefits of PCSK9 inhibition, where the addition of evolocumab to statins in patients with coronary disease resulted in regression of atherosclerotic plaque, as evidenced by intravascular ultrasonography—another testament to the efficacy of achieving lower LDL-C levels [40].

Furthermore, in recent years, a new therapeutic option by inclisiran with only twice-yearly injections has become available. It uses small interfering ribonucleic acid (siRNA)-based technology that degrades PCSK9 mRNA in the liver, inhibiting translation, and thus eliminating the main source of PCSK9 in the circulation. The studies showed that inclisiran can reduce LDL-C by up to 52% and is well-tolerated [41]. Moreover, patient-level analysis of phase III trials suggested the potential benefit of inclisiran in MACE reduction. However, these findings have to be confirmed in larger CV outcome trials with longer follow-ups [42].

In conclusion, PCSK9 inhibitors have revolutionized lipid management by enabling patients to achieve much lower LDL-C levels than previously possible with statins alone, leading to a substantial reduction in cardiovascular risk together with a high intermediate-time safety record [34].

### No Side Effects of Very Low LDL-Cholesterol

The evidence from the available studies collectively supports the conclusion that achieving and maintaining very low LDL-C levels does not result in an increased frequency of side effects, including serious conditions like diabetes, stroke, or cognitive impairment. This information can be reassuring for clinicians aiming for aggressive LDL-C lowering in patients at high risk for cardiovascular events, confirming that the benefits of such an approach outweigh the risks.

In a cognitive function substudy, the occurrence of neurological and neurocognitive events was compared between patients with LDL-C levels below 0.6 mmol/L (23 mg/dL) and those with higher levels. The rates of such events were similar, suggesting that very low LDL-C does not increase the risk of cognitive impairment [43]. The IMPROVE-IT study, which included more than 5000 patients receiving ezetimibe in combination with a statin, achieved LDL-C levels below 1.3 mmol/L (50 mg/dL), with about 1000 patients reaching levels below 0.8 mmol/L (30 mg/dL). Over a follow-up period of 7 years, there was no increased frequency of side effects, including new-onset diabetes, hemorrhagic stroke, or neurocognitive disorders, in these subgroups [44]. This indicates that reducing LDL-C to these low levels is not associated with a higher risk of these conditions.

The previous studies revealed that one of the possible side effects of statin treatment is a 9–12% higher risk of developing diabetes mellitus type 2 (DM2). However, overall benefit of this therapy is greater than the harm (1 new diagnosis of DM2 vs. prevented 5,6 CVD events); therefore, we should still prefer this therapy [45,46]. Furthermore, the risk of developing DM2 was evaluated in PCSK9 inhibitor trials. No excess incidence of diabetes or glucose intolerance was reported in individual studies or in a meta-analysis of 39 randomized controlled trials. This suggests that even with the significant LDL-C reduction caused by PCSK9 inhibitors, there is no associated increase in diabetes risk [47,48]. Moreover, incidences of serious adverse events did not increase over time, which further supports the long-term safety of achieving very low LDL-C levels, as shown by the FOURIER-OLE study [33].

## 5. Strike Early and Strong

Emerging evidence suggests that initiating a potent LLT and achieving an early and substantial reduction in LDL-C shortly after an ACS may be of significant benefit.

The Swedish nationwide cohort study provides compelling evidence supporting the early and significant reduction in LDL-C following MI. The investigation followed 40,607 patients, revealing that those who experienced a larger decrease in LDL-C levels had considerably lower risk ratios for a composite of cardiovascular mortality, MI, and ischemic stroke, as well as other outcomes like all-cause mortality and heart failure hospitalization [49]. Complementing the Swedish study are findings from two other investigations. The first is an analysis of the National Registry of Myocardial Infarction 4, which explored the effect of statins administered within the first 24 h of an acute MI on hospital morbidity and mortality. The study observed that early statin treatment, either continued from prior therapy or newly initiated, was associated with a decrease in the risk of in-hospital mortality and complications compared with no early statin use [50]. The second is a meta-analysis, which included 20 randomized controlled trials, showing a marked reduction in 30-day MI rates, especially when statins were given before rather than after PCI [51].

In the EVOPACS study, evolocumab administered during the in-hospital phase of ACS resulted in a dramatic reduction in LDL-C levels. At 8 weeks, patients treated with evolocumab achieved a mean LDL-C reduction of 40.7%, significantly greater than the placebo group, demonstrating the efficacy of PCSK9 inhibitors in achieving recommended target levels [52]. Likewise, studies such as EPIC-STEMI and VCU-AlirocRT proved that PCSK9 inhibition after ACS leads to a significant, fast, and safe reduction in LDL-C [53,54]. The PACMAN-AMI trial evaluated the impact of alirocumab on coronary atherosclerosis in patients undergoing PCI for acute MI. It showed significant plaque regression in non-infarct-related arteries after 52 weeks compared with a placebo, highlighting the beneficial effects of PCSK9 inhibitors on atherosclerotic changes after ACS [55]. Plaque stabilization and regression were also achieved with evolocumab in statin-treated patients after non-ST-segment elevation myocardial infarction, as shown by the Huygens study [56]. These studies collectively indicate that early initiation of intensive LDL-C lowering therapy, including the use of statins and PCSK9 inhibitors, is associated with favorable changes in plaque characteristics and a lower risk of recurrent MI and MACEs. This supports the strategy of striking early and strong with LLT following ACS to improve long-term cardiovascular health.

In an optimal setting following ACS, it is recommended to progressively intensify LLT, with treatment evaluations occurring after 4 to 6 weeks [5,10,12,14]. Patients starting with high baseline LDL-C levels may need a combination of statins, ezetimibe, and PCSK9 inhibitors—a lipid-lowering triple therapy. Hence, in the ideal post-ACS scenario achieving the recommended LDL-C goal with this regimen could take at least 12 weeks. However, this timeframe for optimizing LLT is particularly critical because the highest risk of recurrent MI occurs within the initial phase post-ACS [57]. Early and potent LDL-C reduction has been associated with improved long-term prognosis, highlighting the necessity for aggressive initial treatment [49,50,51]. As mentioned above, the Acute Cardiovascular Care Society of the European Society of Cardiology has proposed an innovative approach to “strike effective and strong” and begin combination therapy with high-dose statin and ezetimibe directly after ACS [13]. This approach was also outlined by leading cardiologists and consolidated by the BEST (Best Evidence with Ezetimibe/statin Treatment) consensus [58] and Musumeci et al. [7].

However, while the benefits of rapid LDL-C lowering are acknowledged, potential downsides include the high cost of PCSK9 inhibitors, which may not be justifiable without the guarantee of ongoing treatment post-discharge. Moreover, the studies with PCSK9 inhibitors at the time of ACS reduced the burden of an atherosclerotic plaque, but these studies were not powered for clinical outcome, and studies such as FOURIER and ODYSSEY OUTCOMES did not include patients in the acute phase [59].

### 5.1. The Future

Based on experience with an early start of LLT (at that time, mainly statins) even before PCI in ACS patients [51,60], the field anticipates results from forthcoming trials such as EVOLVE-MI (NCT05284747, clinicaltrials.gov), which will test the effects of adding evolocumab to standard care on major cardiovascular events in 4000 post-AMI hospitalized patients. The VICTORION-INCEPTION (NCT04873934, clinicaltrials.gov) trial is assessing the effectiveness of inclisiran, an injectable PCSK9-targeting siRNA, in post-ACS patients with persistently high LDL-C levels despite statin therapy. Moreover, the AMUNDSEN trial (NCT04951856, clinicaltrials.gov) is randomizing patients to receive evolocumab with the first dose given before PCI, aiming to study LDL-C reduction at 12 months with tertiary clinical endpoints. These future studies will further delineate the role of early LDL-C reduction in the acute phase of ACS and help refine guidelines for the management of lipid risk in this critical patient population.

### 5.2. The Pleiotropic Effect of Lipid-Lowering Therapy after Acute Coronary Syndrome

The pleiotropic effects of statins play a crucial role in the management of patients post-acute coronary syndrome (ACS), especially concerning periprocedural cardiac myonecrosis that can follow percutaneous coronary intervention (PCI). These effects are distinct from and may occur independently of their cholesterol-lowering action [61].

Short-term pretreatment with high-dose statins, recommended by European guidelines, has been shown to mitigate the risks associated with PCI—such as distal embolization, side branch occlusion, dissection, or vasospasm—through mechanisms that are not solely related to LDL-C reduction. These benefits occur even within a window too brief to significantly alter lipid profiles, suggesting that the cardioprotective action of statins is attributed to their pleiotropic properties [61].

These pleiotropic properties include improved endothelial function, which is primarily due to increased production of endothelial nitric oxide, leading to enhanced vasodilation and protection against endothelial dysfunction. They also encompass anti-inflammatory effects that inhibit cell proliferation, reduce the aggregation of inflammatory cells, and limit the secretion of pro-inflammatory cellular factors. Furthermore, statins have shown a pronounced impact on plaque stabilization and reduction, an effect considered to be linked to their anti-inflammatory capabilities [62,63,64].

The relationship between statins and platelet function is particularly relevant to ACS. Statins have been associated with reduced platelet reactivity and thrombin generation, which are important factors in the pathogenesis of ACS. This reduction in platelet activity is not solely a consequence of their LDL-C-lowering action but is also a direct effect of the drugs themselves, as demonstrated by improved endothelial nitric oxide synthase (eNOS) function and downregulation of platelet activation markers [63].

In summary, the pleiotropic effects of statins contribute significantly to their cardioprotective role following ACS, offering benefits beyond lipid lowering. These effects include improving endothelial function, reducing vascular inflammation, and mitigating platelet activity, all of which are vital in reducing the incidence of periprocedural cardiac myonecrosis and the overall burden of cardiovascular disease post-PCI [61,62,63,64,65].

Ezetimibe has demonstrated varied effects on platelet activation; while some studies have found it to reduce platelet activation in vitro and as monotherapy [66], other research has indicated no significant impact on platelet function when it is combined with a statin [67,68]. These conflicting results suggest that the role of ezetimibe in modifying platelet activity post-ACS remains to be fully elucidated [69].

PCSK9’s role in vascular inflammation and platelet function is becoming increasingly recognized. Preclinical studies have shown that PCSK9 knockout mice have a reduced incidence of thrombosis and attenuated platelet activation following vascular injury. In humans, elevated PCSK9 levels have been correlated with heightened platelet reactivity even under treatment, suggesting PCSK9’s involvement in platelet activation [69,70,71].

While PCSK9 inhibitors significantly lower LDL-C levels, their direct cardioprotective effects during PCI are not apparent [65]. However, the administration of PCSK9 inhibitors has been associated with reduced platelet activation and a decrease in platelet-driven immunothrombosis [72,73]. Over the longer term, PCSK9 inhibition has been shown to lower plasma levels of platelet activation markers, indicative of a broader influence on platelet function that transcends cholesterol lowering [74]. Inclisiran, a siRNA-based PCSK9 inhibitor, however, has not exhibited any significant effects on platelets [75].

Secondary biomarker analysis of the randomized placebo-controlled multi-center CLEAR Harmony trial showed, besides effective LDL-C lowering, a reduction in inflammatory markers such as CRP, fibrinogen, and Interleukin-6. Hence, bempedoic acid could be a useful treatment option for addressing both residual cholesterol and inflammatory risk after ACS [76].

### 5.3. Novel Strategies for Patients with DM2

GLP-1 receptor agonists (GLP-1RAs) have emerged as influential agents in managing type 2 diabetes mellitus (T2DM), not only for their primary role in glycemic control but also for their beneficial effects on body weight and cardiovascular risk factors. Beyond these known benefits, there is growing evidence to suggest that GLP-1RAs may have favorable effects on lipid profiles, contributing to cardiovascular disease (CVD) prevention [77].

GLP-1RAs have been consistently effective in reducing postprandial lipemia, which is considered a driver for their anti-atherogenic and cardioprotective effects. This reduction in postprandial lipid levels can be mediated by direct activation of GLP-1 receptors, leading to changes in pancreatic hormone secretion, lymph flow, and gastric emptying. Additionally, there may be indirect modulation through central nervous system mechanisms [78,79].

Specific GLP-1RAs, such as semaglutide, have shown efficacy in lowering low-density lipoprotein (LDL) and total cholesterol levels [80]. However, the impact of GLP-1RAs on the lipid profile has yielded mixed results in meta-analyses, with some agents significantly increasing high-density lipoprotein (HDL) concentrations and others showing significant effects on triglyceride levels or no effect on lipid profile at all [80,81,82].

The role of GLP-1RAs in modulating lipid metabolism extends to the liver, with studies indicating a reduction in hepatic steatosis by inhibiting de novo lipogenesis and improving mitochondrial function in hepatocytes. These hepatoprotective effects are mediated through the downregulation of genes involved in lipogenic pathways, contributing to an improved lipid profile [83].

In conclusion, GLP-1RAs contribute to improved CVD outcomes not only through weight management and glycemic control but also potentially through lipid-modulating effects. Although the impact on lipid parameters may vary among the different GLP-1RAs, their role in postprandial lipid metabolism and direct hepatic actions signifies their therapeutic value in managing dyslipidemia associated with T2DM. Further studies are warranted to clarify their lipid-lowering capabilities and the implications for long-term cardiovascular health.

## 6. Strategies for Improvement

The previously discussed real-world data showed that only a small number of patients with CHD achieve the guidelines recommended LDL-C goal. Hence, is it really possible to sufficiently lower LDL-C? Do we have enough tools to achieve the goal?

Achieving the recommended LDL-C goals is indeed possible, as demonstrated by Makhmudova et al. in the “Jena auf Ziel” study. This prospective cohort study initiated early combination therapy with high-dose atorvastatin and ezetimibe in patients with ST-elevation myocardial infarction. Follow-up treatments were escalated with bempedoic acid and PCSK9 inhibitors to achieve recommended LDL-C targets. The study showed that 80% of patients reached their LDL-C targets with the initial combination therapy, and with the addition of either bempedoic acid or PCSK9 inhibitors, all patients achieved LDL-C levels of or below 55 mg/dL (1.4 mmol/L). Moreover, the combined lipid-lowering therapy was well-tolerated with rare side effects [84]. The key is the utilization of combination therapies. This was further supported by Ray et al., who used the SANTORINI data to perform a simulation of adding bempedoic acid (BA) to ezetimibe in the treatment pathway to assess the proportion of patients who might reach the LDL-C goal [85]. BA is a new oral medication that inhibits cholesterol synthesis upstream of the statin pathway [86,87,88]. Without oral therapy escalation, only 1428 (23.1%) of the SANTORINI patients achieved the LDL-C goal. After adding ezetimibe, 2455 (39.7%) patients were predicted to achieve their risk-based LDL-C goal. After the addition of BA, 3677 (59.5%) patients achieved the risk-based LDL-C goal, meaning that the rest of the patients would still need the addition of the PCSK9 antibodies or siRNA therapy targeted against PCSK9, inclisiran [85].

Despite the potential to achieve LDL-C goals, barriers such as lack of adherence, limited access to therapy, and clinical inertia exist [7,13,89,90]. Among the 617 patients in the PALM registry who discontinued statin therapy, 37% did so out of fear of side effects, and 60% because of perceived side effects [90]. Statin-related muscle symptoms were the most common reason for discontinuation also in the IMPROVE-IT trial and a survey from U.S. primary care clinicians [91,92]. Another study also noted that low social status, comorbidities, and polypharmacy are factors that often lead to discontinuation of therapy. Moreover, the rate of muscle-related AE reports excessed only when patients and their doctors were aware that statin therapy was being used and not when its use was blinded, which illustrates the so-called nocebo effect rather than actual pharmacologic side effects [93]. The N-of-1 trial by Wood et al. found that 90% of the symptom burden elicited by a statin challenge was also elicited by a placebo, proving the so-called nocebo effect [94].

Strategies to improve adherence include patient education about the importance of LDL-C management and motivational interviews. Regular lipid monitoring as recommended by guidelines can also help maintain adherence [90].

In the case of real statin intolerance, we still have a lot of other treatment options available. For instance, BA can be used as an alternative. It is beneficial for statin-intolerant patients or for those who have not achieved their LDL-C goals with statin therapy alone. The CLEAR OUTCOMES study found that combined lipid-lowering therapy, which included bempedoic acid, was well-tolerated with rare side effects, suggesting it is a viable option for patients who are unable to tolerate statins. Bempedoic acid’s mechanism of action differs from that of statins, as it requires activation in the liver, thus potentially reducing the risk of muscle-related side effects, which are common reasons for statin discontinuation [86,87,88,95].

PCSK9 antibodies and inclisiran, an injectable medication that lowers LDL-C by 40–52% by enhancing the degradation of PCSK9 mRNA, can also be used when statins are not suitable or lower the LDL-C insufficiently [7,41,96,97]. Furthermore, these substances can improve compliance since it is necessary to inject the PCSK9-antibodies only twice a month (Evolocumab) or once per month (Alirocumab), and inclisiran should be injected two times per year. Moreover, patients on inclisiran therapy have the possibility to be warned about the next injection by the reminder system.

**Figure 1:**
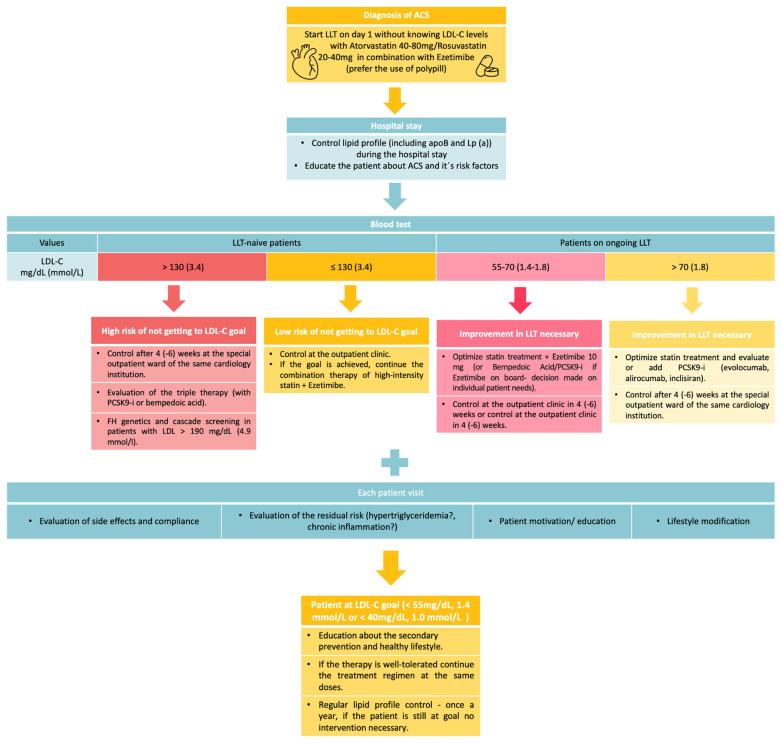
Stepwise proposal by the authors to achieve LDL-C treatment goals in ACS patients. ACS—acute coronary syndrome, LDL-C—low-density lipoprotein cholesterol, LLT—lipid-lowering therapy, Lp (a)—lipoprotein (a), PCSK9-I—proprotein convertase subtilisin/kexin type-9 inhibitor.

Implementation of combination therapy immediately after ACS and subsequent follow-up of the cholesterol profile in the clinic where patients were hospitalized for an ACS several weeks after discharge could also be a way of achieving the recommended therapeutic goal. During this appointment, the LDL-C goal would be evaluated, therapy adjusted if necessary, and the patient educated. This approach could be beneficial, especially for patients with high baseline LDL-C levels who are less likely to achieve their goal with a combination of high-intensity statin and ezetimibe. In her study, Makhmudova showed, that this approach is effective [84]. Data from our clinic seems very promising as well(data not published yet). Furthermore, in our institution, we established a standard operation procedure to increase the proportion of patients reaching the LDL-C treatment goal (Figure 1).

To address clinical inertia, more frequent LDL-C monitoring and polypill approaches combining lipid-lowering therapy with antihypertensive medication can improve medication adherence and reduce the management burden on clinicians. Clinical inertia can also be reduced by embracing health information technology, including clinical decision support systems that can prompt clinicians to adjust treatment according to LDL-C levels [89].

Access issues can be overcome through collaboration between healthcare stakeholders to generate evidence on the value of advanced treatments and develop sustainable models for medical access. This includes the need for stakeholder collaboration to improve patient access to advanced lipid-lowering therapies [89].

In conclusion, it is possible to achieve recommended LDL-C goals with a combination of patient education, medication adherence strategies, immediate combination therapy, alternative therapies for statin intolerance, and systemic changes to address clinical inertia and access issues. The evidence suggests that with appropriate intervention and treatment escalation, achieving the LDL-C goal is achievable for the majority of patients, and the fear of side effects should not be a barrier to effective lipid-lowering therapy [84,85].

## 7. Conclusions

As real-world data show, there is a huge gap in the actual management of dyslipidemia in real-world settings. An unfounded fear should not restrain us from lowering LDL-C as low as possible since it does not show any harm. Moreover, we have enough treatment options to achieve the recommended goal, which seems to be very efficient and safe, and studies are proving that we can achieve the recommended LDL-C goal in almost every patient after ACS. Furthermore, there is a sufficient amount of evidence that the immediate combination of LLT after ACS leads to early LDL-C goal achievement without any safety concerns. On top of it, intensive LLT after ACS led to atherosclerotic plaque stabilization caused not only by the effective lipid-lowering effect of the therapy but also a pleiotropic effect of statins and PCSK9-inhibitors. However, further studies investigating the clinical outcome of novel therapies such as PCSK9-inhibitors and Inclisiran directly after ACS are needed to prove this concept. Moreover, studies focusing on an improvement in the residual risk, such as hypertriglyceridemia or hyperlipoproteinemia (a), after sufficiently reducing LDL-C or non-HDL-c in ACS patients should shed more light on the effective secondary prevention strategies.

## Figures and Tables

**Table 1:** Real-world data on achieving the recommended LDL-C goal of <55 mg/dL (1.4 mmol/L) according to 2019 ESC/EAS Guidelines.

Trial Name	Year of Publication	Country	Participants	Population	LDL-C Goal Achieved
Da Vinci [15]	2021	18 countries in Europe	5888 in total, 2888 in secondary prevention	CVD-secondary care	22.00%
Santorini [8].	2023	14 countries in Europe	9602	High-risk and very high-risk patients *	20.10%
Zuin et al. [16]	2023	Europe, meta-analysis of 7 studies	36,354	History of ACS	12.10%
Khatib et al. [18]	2023	12 hospitals in midwestern USA	5467	1 year after ACS	18.00%
Jain et al. [19]	2023	11 centers in India	575	1 year after ACS	20.90%

ACS—acute coronary syndrome, CVD—cardiovascular disease, USA—United States of America. * CV risk was also assessed centrally based on the information present in the study database according to SMART, Framingham, or SCORE risk score systems per 2019 ESC/EAS guideline criteria [20,21].
